# A high-fidelity prototype of a sterile information system for the perioperative area: OR-Pad

**DOI:** 10.1007/s11548-022-02787-w

**Published:** 2022-11-12

**Authors:** C. Ryniak, S. M. Frommer, D. Junger, S. Lohmann, M. Stadelmaier, P. Schmutz, A. Stenzl, B. Hirt, O. Burgert

**Affiliations:** 1 School of Informatics, Research Group Computer Assisted Medicine (CaMed), Reutlingen University, Reutlingen, Germany; 2 Department of Urology, University Hospital Tübingen, Tübingen, Germany; 3 Faculty of Medicine, Department of Anatomy, Institute for Clinical Anatomy and Cell Analytics, Eberhard Karls University Tübingen, Tübingen, Germany

**Keywords:** OR-Pad, Sterile information system, User interface, High-fidelity prototype, Usability test, Perioperative area

## Abstract

**Purpose:**

Supporting the surgeon during surgery is one of the main goals of intelligent ORs. The *OR-Pad* project aims to optimize the information flow within the perioperative area. A shared information space should enable appropriate preparation and provision of relevant information at any time before, during, and after surgery.

**Methods:**

Based on previous work on an interaction concept and system architecture for the sterile *OR-Pad* system, we designed a user interface for mobile and intraoperative (stationary) use, focusing on the most important functionalities like clear information provision to reduce information overload. The concepts were transferred into a high-fidelity prototype for demonstration purposes. The prototype was evaluated from different perspectives, including a usability study.

**Results:**

The prototype’s central element is a timeline displaying all available case information chronologically, like radiological images, labor findings, or notes. This information space can be adapted for individual purposes (e.g., highlighting a tumor, filtering for own material). With the mobile and intraoperative mode of the system, relevant information can be added, preselected, viewed, and extended during the perioperative process. Overall, the evaluation showed good results and confirmed the vision of the information system.

**Conclusion:**

The high-fidelity prototype of the information system *OR-Pad* focuses on supporting the surgeon via a timeline making all available case information accessible before, during, and after surgery. The information space can be personalized to enable targeted support. Further development is reasonable to optimize the approach and address missing or insufficient aspects, like the holding arm and sterility concept or new desired features.

**Supplementary Information:**

The online version contains supplementary material available at 10.1007/s11548-022-02787-w.

## Introduction

Access to relevant information in the perioperative area may support surgeons and their team during surgery in the operating room (OR). The visualization equipment within present ORs varies from highly integrated, networked ORs (e.g., KARL STORZ OR1™, Stryker iSuite, Brainlab Digital O.R.) with multiple monitors to less modern ORs with maybe just one smaller monitor. Depending on the intervention and available equipment, monitors may visualize the endoscopic video, radiological images, navigation information, or electronic patient records. In many cases, no display options are available near the operating table [1] or monitors are placed at positions requiring larger head movements resulting in unergonomic setups. Supportive materials, like notes, can hardly be used in the OR [2]. Moreover, the surgeon often cannot interact with the visualization and needs assistance [3]. The application-oriented research project *OR-Pad* [2] aims on improving the information flow in the perioperative area. Via a sterile-packed tablet PC, clinical information is displayed close to the surgeon and can be controlled by the surgeon via touch. To enable context-aware assistance, the information can be prepared preoperatively, displayed and supplemented intraoperatively, and also used postoperatively for documentation (see interaction concept and system architecture [2]). A situation recognition is used to minimize the number of necessary interactions by automatically displaying preselected information according to the actual situation in the OR.

The relevance of such context-aware assistance is demonstrated by a large number of works in this field. For example, Franke et al. [4] address a surgical working environment for ENT surgery to enable context-aware assistance like information presentation. Depending on tracking and processing of the surgical situation, automated selection of appropriate video sources (e.g., endoscopic image or PACS viewer) is done for two displays. Katić et al. [5] propose a system for automatically filtering available information based on the recognized phase of laparoscopic liver and gallbladder surgery. Based on the surgical phase, an appropriate visualization in augmented reality, showing the tumor direction, resection line, or vital structure, is chosen. Similarly, Schreiber et al. [6] show a concept for consistent and prioritized presentation of surgical information for FESS surgery. One display was used for essential information; less important information was shown on a second display, both arranging several information entities (e.g., endoscope, navigation, or PACS). Stauder et al. [7] present a workflow-driven system for breast cancer surgery that combines live imaging sources and DICOM files. The sterile draped display is in reach of the surgeon so that the sources can dynamically be switched via gestures.

The displaying approaches differ by provided information, e.g., Franke et al. [4] select an appropriate video source, whereas Katić et al. [5] adapt the augmented reality visualization. The *OR-Pad* [2] has the goal to enable a shared information space for preparation, intervention, and follow-up, covering all available information from HIS and PACS as well as personal information. To avoid information overload and distraction, the information needs to be displayed in a structured and clear form, being easily accessible and usable without additional cognitive load for the surgeon. Our user interface approach is based on a timeline, inspired by other systems visualizing electronic health records [8]. This paper describes the user interface concept consisting of a timeline as the main element combined with other features, like annotating elements in an image, for targeted assistance. Via prototypical implementation and functional as well as preclinical evaluation of the prototype, the feasibility of the *OR-Pad* vision, as well as the usability of the visualization approach, is shown.

## Methods

The *OR-Pad* system was developed within four development cycles using a user-centered design process (see [2]). The first user interface focused on separated functionalities in form of independent, supporting features (e.g., section for patient information, notes, or camera source). Expert interviews revealed that the structure and linking of functionalities should be reconsidered. The second approach addressed a process-based view by providing different functionalities depending on the pre-, intra-, or postoperative phase within the OR. Information was provided via a media library, and features were connected to the information (e.g., draw in an image). A usability test showed very good results but made an overload of information apparent. The third iteration focused on supporting the surgeon via access to information and improvement of the information flow. Based on that, the requirements of the final prototype were specified (see Online Resource 1) and the *OR-Pad* system was redesigned.

### Interaction concept and system architecture

Our previous work [2] depicts the interaction concept and system architecture of the *OR-Pad* system. The interaction concept consists of the idea of a mobile and an intraoperative mode to support the surgeon before, during, and after surgery. The mobile app can be used on a smartphone or laptop for preparation and follow-up, the intraoperative app on a tablet during the intervention. Before surgery, the surgeon can view upcoming interventions and their available case information. Important information can be highlighted, new information be added, and context-aware support be managed. During surgery, the surgeon can access all case information and the prepared content. Context-relevant content is displayed depending on the surgical phase. Information can be filtered, edited, and added. After surgery, the surgeon can review and edit the gathered information.

The client–server system architecture described in [2] was implemented as a progressive web app. The clients are using responsive user interfaces and hardware access to view and add information. The server is responsible for data management and connecting external systems like HIS and PACS to retrieve patient data. A situation recognition system is connected to enable context-sensitive information display. For the communication with these systems, we used the established standards FHIR and DICOM.

### User interface concept

**Fig. 1:**
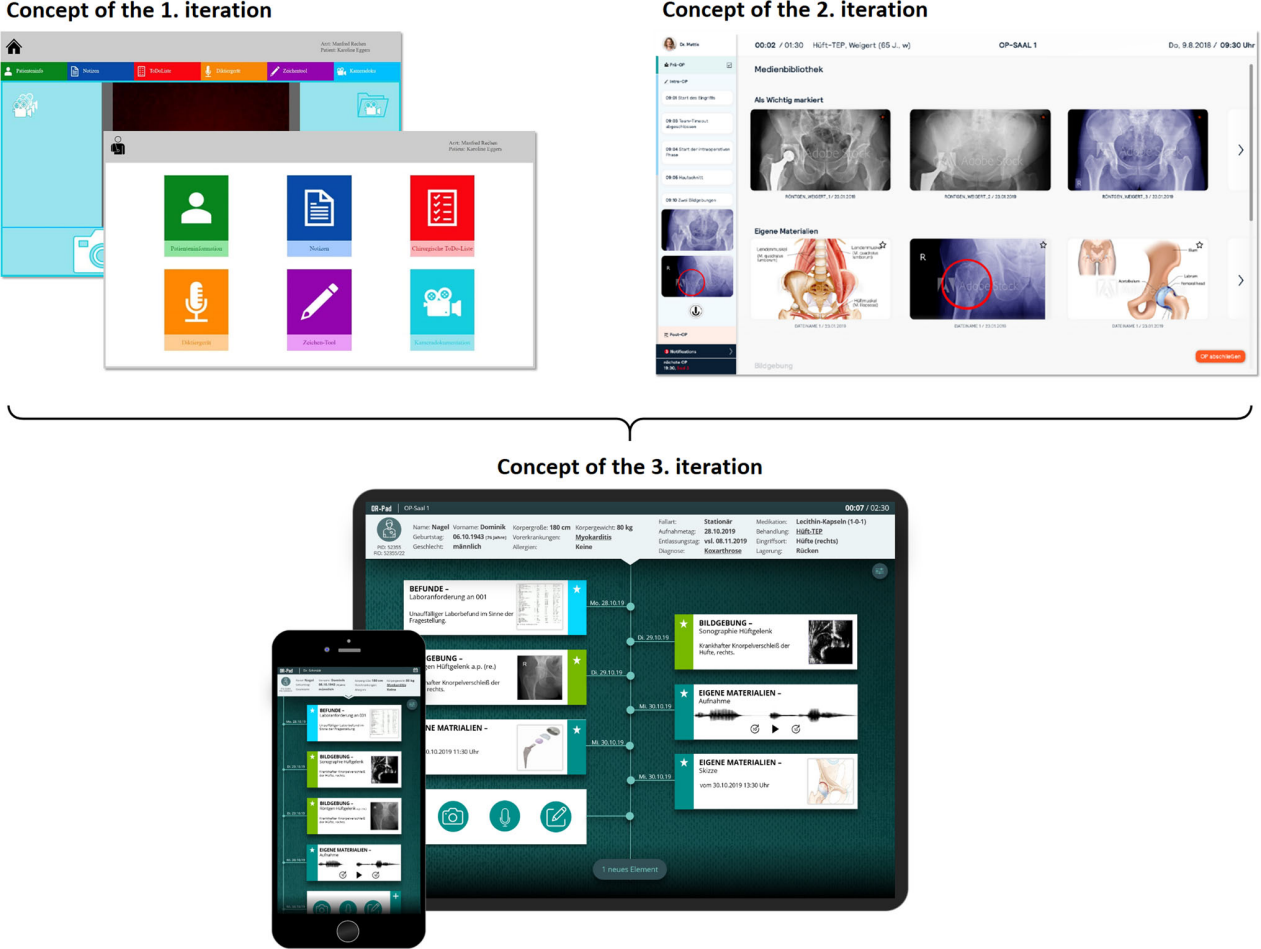
Evolution of the user interface concepts of the *OR-Pad*. The concept of the third iteration combines the positive features of the previous concepts

The user interface concept combines the positive features of previous approaches [2] (see Fig. 1). Fast access to interaction functionalities, like recording voice memos or taking pictures, was taken from iteration one. The data-centric approach for viewing and editing clinical information via connected features was taken from iteration two. This led to a new concept for pre-, intra-, and postoperative use, consisting of a mobile and intraoperative mode that share the same design concept.

The central element is the timeline which displays the available case information of the intervention chronologically, according to the recording times, and categorizes this information by color coding according to its type, e.g., imaging. In addition, at the very bottom of the timeline, a function field is visible which can be used to create new materials, such as a photograph/video, a note, or a voice recording. By clicking on a timeline entry, the corresponding information can be viewed full-screen and, depending on the medium, their view can be modified, e.g., scrolling through the layers of CT images. A filter option in the upper right corner allows individualization of the view and also facilitates quick retrieval. In the upper area of the user interface, the patient and procedure information of the current intervention is displayed. In the intraoperative mode, the status bar also shows the current OR, the scheduled duration, and the time since the start of the surgery. The two modes also differ in the login and first view before getting to the timeline view.

The vector-based graphics software Adobe XD was used to transfer the graphical concept into a clickable prototype which was shown to our clinical partners of the University Hospital Tübingen (see [2]). After the good feedback from our clinical partners, the implementation of the whole system started.

### Implementation and evaluation

#### Prototypical implementation

In the fourth iteration, the developed concepts and approaches described before were realized within a high-fidelity prototype. We simulated a clinical software infrastructure by realizing the HIS, PACS, and their communication interfaces. The open-source FHIR server Aidbox.Dev was used as the HIS and the DICOM server Orthanc as the PACS. Seven de-identified test data sets from orthopedics were used. For retrieval of the stored data, we used the libraries fhir-kitclient and orthanc-client. All clinical information is therefore organized within FHIR and DICOM resources for standardized handling. For testing the situation-related displays, the situation recognition was simulated to enable an independent system that works without dependencies to ensure that all test participants are using the same environment and setting. The infrastructure of the *OR-Pad* system consists of a virtual machine (VM) that serves as the *OR-Pad* server and contains the *OR-Pad* database (MongoDB) and PACS software, an additional physical computer that represents the *OR-Pad* HIS server on which the HIS software Aidbox.Dev is running, and the clients. The communication between clients and server is realized by web sockets.

The *OR-Pad* application was implemented using JavaScript, HTML, and CSS. For the *OR-Pad* demo prototype, an iPad Pro, a Samsung Note, and a Windows 10 PC were used as end devices.

#### Test setup

The developed prototype was integrated into the research OR at Reutlingen University. The evaluation was split into a functional and preclinical evaluation. In the functional evaluation, the requirements were compared to the actual functionalities by executing the *OR-Pad* demo prototype for the various use case scenarios. Furthermore, feedback from clinical partners was included via expert interviews with our project partners Prof. Stenzl from urology and Prof. Hirt from anatomy of the University Hospital Tübingen.

In the preclinical evaluation, which served as our final evaluation, the usability was tested with clinicians, the functionalities were examined, and a quantitative assessment was done via questionnaires. The prototype was running on a Samsung Galaxy Note 10 Lite with 6.7" and an Apple iPad Pro 3 with 12.9". Both clients communicated with the *OR-Pad* server. The study was conducted with twelve surgeons and assistant doctors from four different clinics in Germany and different disciplines. A neutral meeting room was provided as a test environment. The study was conducted separately with each test person. In the beginning, each participant received a short introduction in which the aim of the system and the planned application were explained. After the introduction, the test person had about ten minutes to familiarize with the system. Subsequently, the participant got typical tasks for the system for the scenarios of preparation and follow-up as well as the performance of an intervention, to solve independently with the help of the mobile and intraoperative app. The clickstream (record the number of clicks) was logged and the person was asked to speak their thoughts out loud (think-aloud method). Audio and screen recording was done.

To be able to evaluate the usability of the system quantitatively, the participant filled out a questionnaire after completing the tasks, which is based on the System Usability Scale (SUS). This is a technology-independent questionnaire comprising ten scale questions to evaluate the subjectively perceived usability in points. The SUS score is calculated from the results of the SUS questionnaire by coding the categories with values from 0 for rejection of the system to 4 for acceptance of the system. These are added and multiplied by 2.5 to allow for a SUS score between 0 and 100, with 100 being the top rating. To evaluate the range of functions, additional open interview questions were asked. These were intended to identify superfluous or missing functionalities as well as further ideas for areas of application.

## Results

The *OR-Pad* application was realized as a client–server application and implemented as a progressive web app with various functionalities and operating options. For technical details on the interaction concept and system architecture, please refer to our pre-work [2], as this article focuses on the graphical user interface. The frontend was developed in German; relevant aspects are described in the text.

### High-fidelity prototype

#### Login and intervention overview

**Fig. 2:**
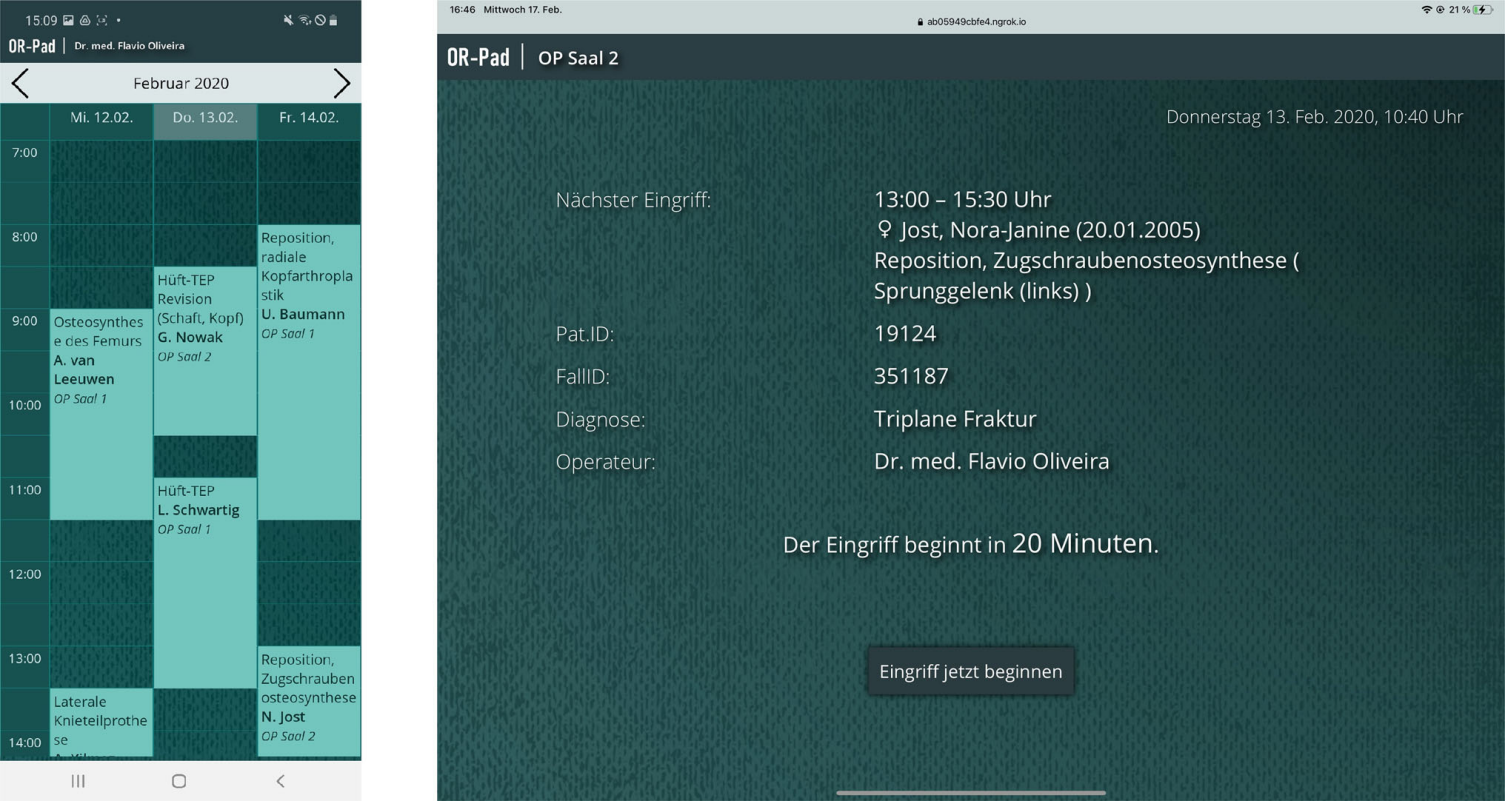
Calendar of the mobile mode (left) and overview of the upcoming intervention of the intraoperative mode (right)

After starting the application, the user is asked to log in. After login, the mobile application shows a calendar view of past and future interventions which are assigned to the registered user (see Fig. 2 left). The user can look at detailed information about the intervention by clicking on it, so that more information of the particular intervention is dynamically loaded from the clinical systems. In the intraoperative application, an overview of the upcoming surgery is generated (see Fig. 2 right). Information such as the time of the intervention, the patient, or the type of surgery is displayed. The user can check whether the correct intervention is indicated and start it via a button. Further information is loaded to be displayed.

#### Main view

**Fig. 3:**
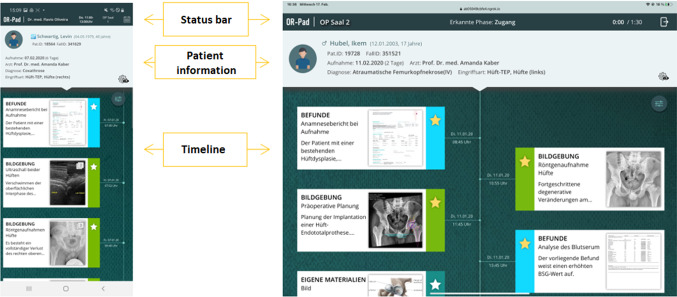
Main view of the *OR-Pad* application (left mobile and right intraoperative mode)

After selecting or starting an intervention, the user is taken to the main view (see Fig. 3). This consists of three components: a general status bar at the top, the patient information, and the timeline. The status bar is visible (once logged in) in every view except in the full-screen view of a material. In the mobile application, the status bar consists of the *OR-Pad* logo, the logged-in surgeon, the time slot of the selected procedure, the location, and a calendar button. In the intraoperative application, the status bar consists of the *OR-Pad* logo, the logged-in OR, the actual phase provided by the situation recognition, the progress of the surgery including the scheduled time, and an exit button. If material is available for the recognized phase, this is indicated by the eye symbol in the status bar and it can be opened via click. The patient information component consists of the name, birthday, and age as well as the patient and case ID of the patient. In addition, the day on which the patient was admitted (including the days since then), attending surgeon, diagnosis, and type of intervention are displayed. The cogwheel symbol at the bottom right provides an overview of the material phase assignment.

The central component of the application is the timeline. It displays all information chronologically, arranged along a vertical timeline. The arrangement is according to the date of creation from old (top) to new (bottom). It also has functions for creating own material at the end of the timeline and the possibility to filter the information. Timeline entries are visualized in containers. A container consists of the name of the category to which the material is assigned (findings, imaging, or own materials), the name of the material, and a text. Next to it is a thumbnail. At the edge of the container, a colored bar (category of the material) with a star symbol (mark as ‘Important’) is visible. Clicking on the container takes the user to the view of the clicked material. By doing this, all information regarding this material is dynamically loaded from HIS and PACS.

At the top of the timeline in the right corner is the filter button to filter materials according to their category. In the drop-down menu, the user can select ‘All’, the three categories ‘Findings’, ‘Imaging’, and ‘Own materials’ as well as materials marked as ‘Important’. At the very bottom of the timeline, new materials can be created via uploading a file, taking a photograph/video, recording a voice recording, or creating a text note, which are added to the timeline.

#### Material view

**Fig. 4:**
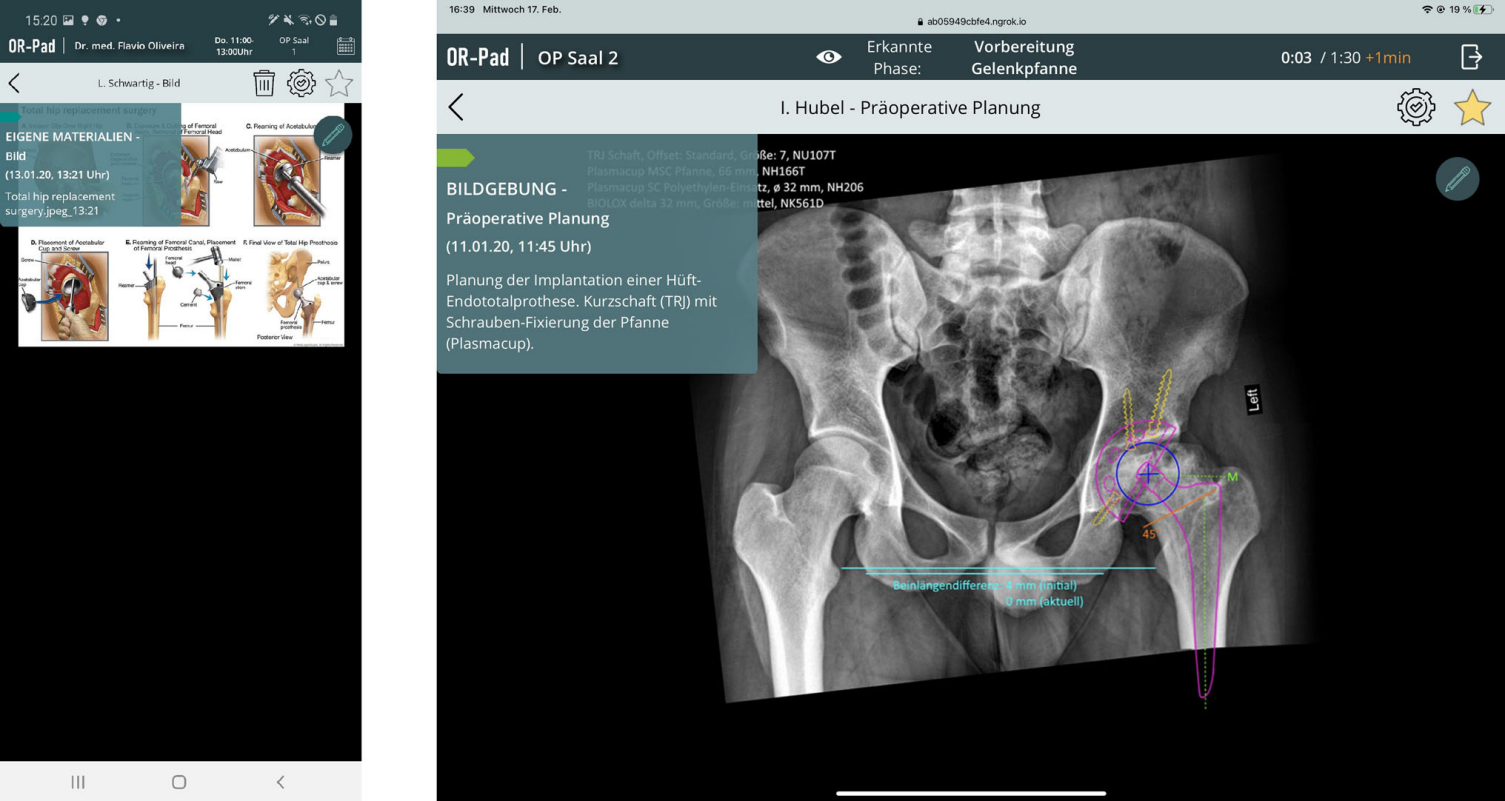
Material view (left mobile and right intraoperative mode)

**Fig. 5:**
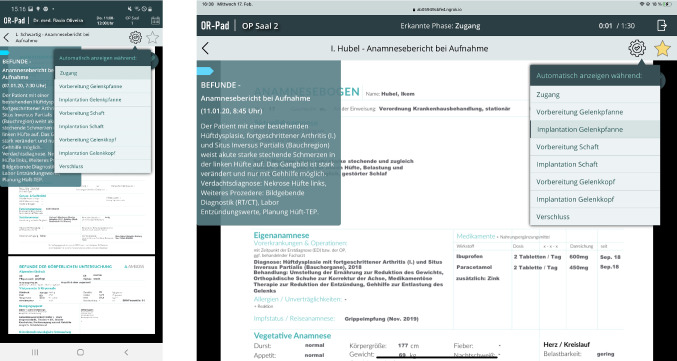
Drop-down menu showing the surgical phases to assign the material (left mobile and right intraoperative mode)

**Fig. 6:**
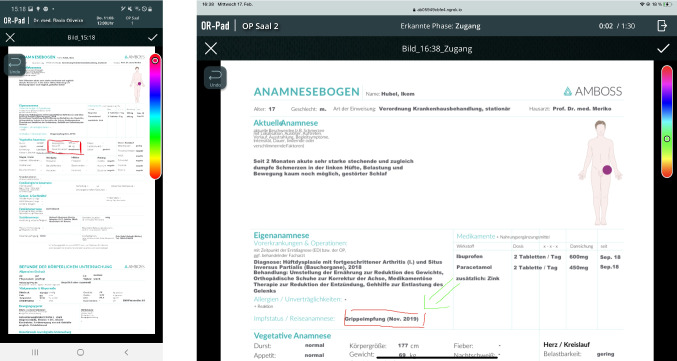
Editing mode to draw into the material (left mobile and right intraoperative mode)

By selecting an entry in the timeline or clicking on the eye symbol, the detailed view of the material is shown (see Fig. 4). Next to the material itself, an overlay with the most important meta-data and the color marking of the category is shown. By clicking on the material, it is displayed in full screen and zooming is possible. The bar above the material contains different elements. The arrow symbol on the far left takes the user back to the timeline view. In the middle is the name of the material, on the far right a bin, cogwheel, and star icon are shown. With the bin symbol, the user can delete materials that belong to the category 'Own materials.' By clicking on the cogwheel icon, a drop-down menu opens in which the user gets a list of surgical phases (see Fig. 5). The user can now select during which phase the selected material should be provided. The star symbol allows the user to mark material as 'Important.' Within the material view, the material can be edited by clicking the pencil icon. In edit mode, the user can draw onto the material (see Fig. 6).

#### Interaction

The *OR-Pad* application focuses on touch interaction, although operation on a PC/laptop via mouse is also possible. Familiar interactions used include ‘tap’ (ordinary touch movement, comparable to a 'click'), ‘flick’ (swipe movement), ‘pinch’ (pull two fingers together), and ‘spread’ (pull two fingers apart). In addition, ‘scroll’ to move content or representations is possible.

### Evaluation

#### Functional evaluation

Online Resource 1 summarizes the evaluation results of the vision, goals, and requirements of the *OR-Pad* system (more details on the evaluation method are found in Online Resource 2). Four of the 5 visions were fulfilled completely. The *OR-Pad* enables the creation and transfer of materials within the perioperative area. Furthermore, the materials and case information can be viewed (quick and uncomplicated access) and relevant information be highlighted. The *OR-Pad* supports the surgeon with context-relevant information during surgery. Nine of the 10 goals were met. In terms of functional requirements, 57 out of 58 were fulfilled. Seventeen of 26 non-functional requirements were met.

For demonstration purposes, the prototype only accesses demo data, a demo situation recognition, a demo HIS, and a demo user administration, so concrete requirements for clinical use with real systems should be collected and implemented. Partially or unfulfilled requirements are mainly due to the prototypic integration in a hospital information system infrastructure and open issues regarding the holding arm and related sterility. Under the assessment of the application exclusively as a demo prototype, the result of the functional evaluation turned out good overall. The expert interviews were very positive encouraging the prototypic implementation.

#### Preclinical evaluation (usability study and functional scope)

Within the usability study, the most striking task in the evaluation of the clickstreams was the assignment of a material to a surgical phase so that the material can be accessed quickly in this situation. On average, the number of clicks required by the test participant for this task was 4.78 clicks higher than the number of required clicks. Only three subjects needed a minimum of three clicks to complete the task. The think-aloud protocol revealed that the phases are too hidden and the process of assigning was unclear to the participants. The icon for the button was described as not intuitive. When the test persons worked on the tasks, it also became visible in the clickstreams that there were minor problems when working on a material. There was an average deviation of 1.55 clicks between the actual and target values for the number of clicks needed to solve the task. One outlier was particularly noticeable here, which required twelve instead of two clicks to solve the task. The interaction in the material view to start and end the full-screen mode was often described as unclear or unnecessary and also led to the outlier in the clickstream data.

During the usability study, all participants could access case information for interventions fast and without any complications. They quickly found the materials they were looking for in the timeline. The participants were reaching every function or information within a short time and minimum amount of clicks. Furthermore, all of the test persons were noting that they think the design of the graphical user interface is good. Despite this positive feedback, also suggestions referred to the timeline arose: One clinician mentioned that the chronological order of the information within the timeline is less interesting than sorting the information according to the course of the intervention, i.e., surgical phases in which the information is needed. Another participant proposed to optically distribute the timeline into sections to differentiate between information that was added before, during, and after the intervention.

**Fig. 7:**
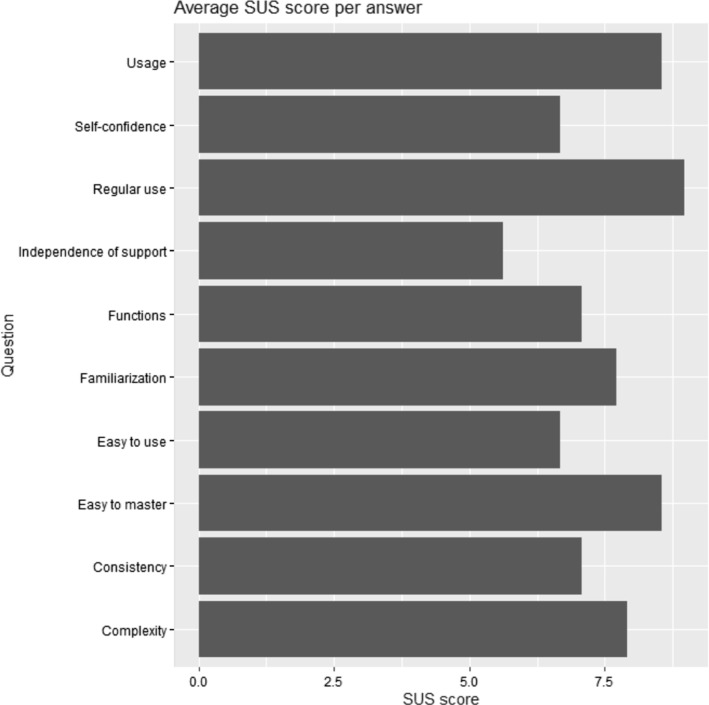
Average SUS score per answer. A rating from 0 to 10 points is possible for each question, with 10 being the best rating

The average SUS score achieved is 74.79. This places usability close to the upper quarter of the scale and can be rated as good. An overview of the average score per question is listed in Fig. 7. However, the usability of the prototype shows further potential for improvement. Many test persons described the scope of the system as too large for everyday surgery and better suited for more complex interventions. Nevertheless, many suggestions for extensions were made, some of which are only needed in specific disciplines.

For further development of the system, this results in several points that need to be addressed. The user-friendliness of the system should be adapted, especially concerning the two particularly conspicuous tasks (i.e., material phase assignment and material view/edit). Furthermore, the suggestions referring to the timeline visualization should be investigated. Likewise, the development of new functions is possible to support surgeons even better and to expand the system's areas of application. The most common wish was a dictation function. In addition, it is possible to expand the intraoperative mode, for example, by implementing a stopwatch or timer that can be started when time-critical events such as the clamping of an artery take place. Another suggestion was to connect to other devices such as an endoscope or navigation system to display the information on the screen near the patient. There were also suggestions for expansion in terms of follow-up, such as the semi-automatic creation of OR reports based on a checklist or similar.

## Discussion

**Fig. 8:**
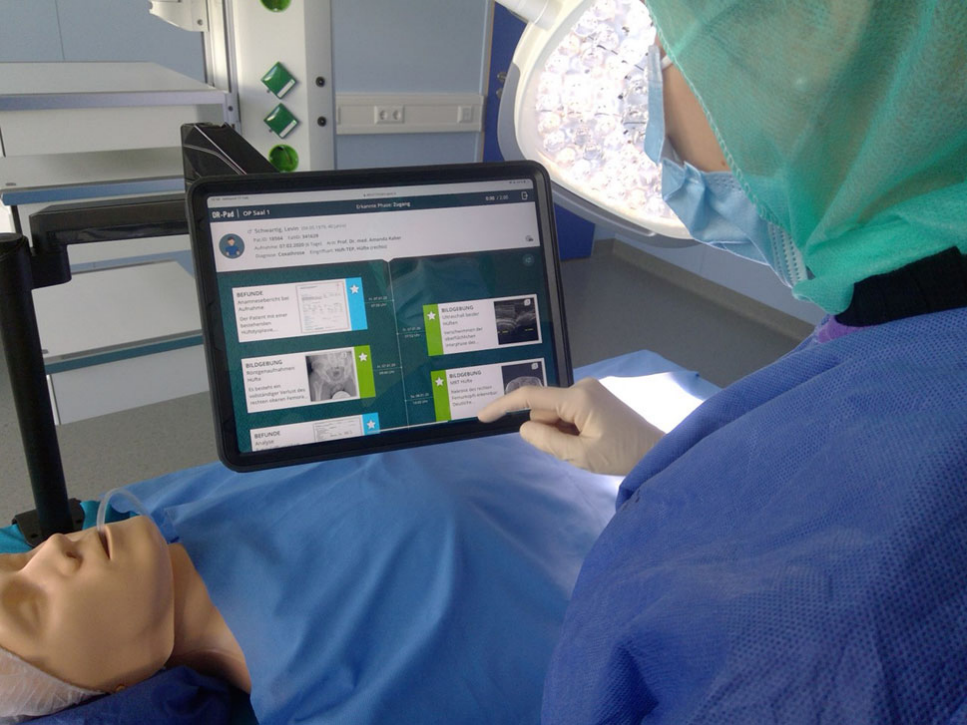
Intended intraoperative use case for the *OR-Pad* (information near to the patient, sterile interaction), demonstrated in the research OR in Reutlingen University

With the *OR-Pad*, the surgeon can select information preoperatively and has it available intraoperatively on a tablet close to the patient (see Fig. 8). In contrast to Franke et al. [4], in which information presentation is not personalized yet, the surgeons can form their information space by themselves which allows a user-adapted visualization in the *OR-Pad*. Similar to Stauder et al. [7], which use gestures for direct interaction, sterile interaction with the system via touch is possible for the surgeon. The surgeon can access relevant images and other information via an intuitively designed interface, including a timeline. In comparison with the concept of the second iteration, the timeline element puts all the information on a case in a chronological context and thus enables an overview and faster retrieval by the surgeon, instead of only providing a simple cluttered media library. Integrated features, like marking a tumor within a radiological image, are linked directly to the medium as before. The included filter function enables information to be found quickly and reduces information overload. Materials for a surgical phase (provided via the situation recognition simulation) can be accessed with one click. The *OR-Pad* reduces the interaction in the intraoperative mode by features like filtering, highlighting, and automatic provision. The shared information space allows the user to have all information available regardless of whether the mobile or intraoperative mode is used.

The evaluations showed good results and confirmed the vision and concepts. Usability issues occurred during annotation (drawing) in medical images and during assigning materials to a surgical phase. Two participants also suggested optimizations of the timeline for a better overview and faster retrieval of information. Changing the order of the timeline from chronologically to phase-dependent may be an option, especially for the intraoperative mode. This would allow that information needed in the actual surgical phase can be found quickly. On the other hand, it would lead to a more time-consuming material phase assignment before the intervention and un-assigned information may be overseen. Another option is the distribution of the timeline into the sections ‘pre’, ‘intra’, and ‘post’ for better differentiation of, e.g., preoperative and intraoperative recorded images. Because of different expectations, an optimal solution needs to be identified in further research. Configurability may be an approach to adapt the timeline visualization to different needs and preferences.

Future topics are the integration of new use cases, the provision of video data streams (e.g., endoscope), and the implementation of secure authentication. Desired functionalities, such as communication with other departments from the OR, e.g., in the form of a secure chat, a dictation function, or feedback to the situation recognition, can also be suitably integrated. Other possible ideas for expanding the system include supporting patient education with various materials and streaming the video image outside of the OR. Regarding the large range of functions, customizability of the interface and the functions would be one way to prevent the system from appearing too cluttered and still be applicable to all disciplines and preferences while also avoiding information overload. Topics that were not considered sufficiently or at all are IT security (data protection), the adaption of communication interfaces to the hospital’s systems, tests in a clinical environment via a clinical evaluation, and consideration and evaluation of the system as a medical device. A concept for holding arm and sterility already exists (see Fig. 8) but needs to be further optimized by addressing open issues, e.g., a sterile cover tailored to the holding arm and tablet.

## Conclusion

We are currently incorporating the evaluation results in the next version of the *OR-Pad* application. Afterward, the application will be tested in a clinical setting to better assess the applicability in the OR and the relevance of the information and functionalities. For this, it could also be considered to exchange the simulation of the situation recognition with the basic framework prototype of the situation recognition system from [9]. Therefore, an SDC interface was conceived and realized to provide the context data of the situation recognition to the *OR-Pad* [10].

## Supplementary Information

Below is the link to the electronic supplementary material.

Supplementary file1 (DOCX 33 kb)

Supplementary file2 (DOCX 41 kb)
