# Supplementary material for: A high-fidelity prototype of a sterile information system for the perioperative area: OR-Pad
Source: Int J Comput Assist Radiol Surg. 2022 Nov 12;18(3):575–85. doi: 10.1007/s11548-022-02787-w (PMC9939502; doi:10.1007/s11548-022-02787-w)
Supplement: Supplementary file 1 — Supplementary file1 (DOCX 33 kb) [file 11548_2022_2787_MOESM1_ESM.docx]

**Supplementary Material for the article “A high-fidelity prototype of a sterile information system for the perioperative area - OR-Pad” published in the "International Journal of Computer Assisted Radiology and Surgery"**

C. Ryniak¹*, S. M. Frommer¹*, D. Junger¹, S. Lohmann¹, M. Stadelmaier¹, P. Schmutz¹, A. Stenzl^2^, B. Hirt^3^, O. Burgert¹

¹Reutlingen University, School of Informatics, Research Group Computer Assisted Medicine (CaMed) Reutlingen, Germany

^2^University Hospital Tübingen, Department of Urology, Tübingen, Germany

^3^Eberhard Karls University Tübingen, Faculty of Medicine, Department of Anatomy, Institute for Clinical Anatomy and Cell Analytics, Tübingen, Germany

*The first two authors contributed equally to this paper.

**Corresponding Author**

Denise Junger, ORCID: 0000-0002-7895-3210

Reutlingen University, School of Informatics, Research Group Computer Assisted Medicine (CaMed), Reutlingen, Germany

Contact: denise.junger@reutlingen-university.de, Tel.: +49 (0)7121 – 271 4090

**Online Resource 1: Requirements analysis & Functional evaluation (summarized version)**

**Table 1** Requirements analysis & Functional evaluation of the *OR-Pad* system (summarized version). V = Vision, G = Goal, F = Functional requirement, N = Non-functional requirement, OR = Operating room, HIS = Hospital information system. More details are found in Online Resource 2.

| ***No.*** | ***Vision, goal or (non-) functional requirement*** | ***Result*** |
| --- | --- | --- |
| **/V01/** | *The OR-Pad system offers fast, uncomplicated access to available case information of an intervention.* | **Fulfilled** |
| **/V02/** | *The OR-Pad system enables the creation and facilitates the transfer of materials into and out of the OR.* | **Fulfilled** |
| **/V03/** | *The OR-Pad system displays materials and case information close by the surgeon during an intervention.* | **Partially fulfilled** |
| **/V04/** | *The OR-Pad system allows to view materials and case information and to highlight relevant aspects in them.* | **Fulfilled** |
| **/V05/** | *The OR-Pad system supports the surgeon during an intervention with context-relevant information.* | **Fulfilled** |
| **/G01/** | *To provide uncomplicated access to available case information of an intervention, the OR-Pad system should realize a connection to various HIS and thus enable bundled access to the available case information.* | **Fulfilled** |
| **/G02/** | *To provide quick access to available case information of an intervention, the OR-Pad system should only display information related to the case and thus reduce the amount of data.* | **Fulfilled** |
| **/G03/** | *To facilitate taking materials into the OR, the OR-Pad system should provide a mobile application for preparation that allows files, such as notes, sketches, voice recordings, etc., to be added and saved for later use.* | **Fulfilled** |
| **/G04/** | *To facilitate the taking of materials out of the OR, the OR-Pad system should provide a mobile application on a tablet during the intervention that allows the creation and storage of files, such as camera screenshots, sketches, voice recordings, etc., for later use.* | **Fulfilled** |
| **/G05/** | *To enable the creation of materials, the OR-Pad system should provide tools for voice recordings, text notes, and camera recordings, as well as allow to upload files to provide an appropriate form of input depending on the situation.* | **Fulfilled** |
| **/G06/** | *To display materials and case information near the surgeon during an intervention, the OR-Pad system should be mounted in the vicinity of the OR site via a suitable holding construction and thus be operable by the surgeon.* | **Partially fulfilled** |
| **/G07/** | *To enable a view of materials and case information, the OR-Pad system should include appropriate tools to display this data, making it accessible to the user.* | **Fulfilled** |
| **/G08/** | *To enable highlighting of relevant aspects in materials and case information, the OR-Pad system should provide appropriate tools for modification, making the view of the data changeable for the user.* | **Fulfilled** |
| **/G09/** | *To support the surgeon with context-relevant information during an intervention, the OR-Pad system should offer the user the possibility to determine which material or case information should be displayed at which surgical phase.* | **Fulfilled** |
| **/G10/** | *To support the surgeon with context-relevant information during an intervention, the OR-Pad system should visualize the temporal progress and thus provide the user with feedback on whether the schedule can be adhered to.* | **Fulfilled** |
| **/F01/** | *The OR-Pad system shall allow the user in the mobile application to authenticate himself.* | **Fulfilled** |
| **/F02/** | *The OR-Pad system shall allow the user in the mobile application to select an intervention.* | **Fulfilled** |
| **/F03/** | *The OR-Pad system shall allow the user in the mobile application to prepare an intervention.* | **Fulfilled** |
| **/F04/** | *The OR-Pad system shall allow the user in the mobile application to select a case information or material.* | **Fulfilled** |
| **/F05/** | *The OR-Pad system shall allow the user in the mobile application to view case information and materials.* | **Fulfilled** |
| **/F06/** | *The OR-Pad system shall allow the user in the mobile application to select tools to edit the case information and materials.* | **Fulfilled** |
| **/F07/** | *The OR-Pad system shall allow the user in the mobile application to customize case information and materials with the provided tools.* | **Fulfilled** |
| **/F08/** | *The OR-Pad system shall allow the user in the mobile application to mark case information and materials as "important".* | **Fulfilled** |
| **/F09/** | *The OR-Pad system shall allow the user in the mobile application to assign case information and materials to a certain phase.* | **Fulfilled** |
| **/F10/** | *The OR-Pad system shall allow the user in the mobile application to see the overview of the material-phase-assignment.* | **Fulfilled** |
| **/F11/** | *The OR-Pad system shall allow the user in the mobile application to filter case information and materials by category.* | **Fulfilled** |
| **/F12/** | *The OR-Pad system shall allow the user in the mobile application to create a new material via a voice recording, text note, or camera recording and to upload files.* | **Fulfilled** |
| **/F13/** | *The OR-Pad system shall allow the user in the mobile application to log out.* | **Fulfilled** |
| **/F14/** | *The OR-Pad system shall allow the user in the intraoperative application to authenticate with the OR credentials.* | **Fulfilled** |
| **/F15/** | *The OR-Pad system shall allow the user in the intraoperative application to register the tablet for the corresponding OR in which it is to be attached.* | **Fulfilled** |
| **/F16/** | *The OR-Pad system shall allow the user in the intraoperative application to log out.* | **Fulfilled** |
| **/F17/** | *The OR-Pad system shall allow the user in the intraoperative application to start an intervention.* | **Fulfilled** |
| **/F18/** | *The OR-Pad system shall allow the user in the intraoperative application to select a case information or material.* | **Fulfilled** |
| **/F19/** | *The OR-Pad system shall allow the user in the intraoperative application to view case information and materials.* | **Fulfilled** |
| **/F20/** | *The OR-Pad system shall allow the user in the intraoperative application to select tools to edit the case information and materials.* | **Fulfilled** |
| **/F21/** | *The OR-Pad system shall allow the user in the intraoperative application to customize case information and materials with the provided tools.* | **Fulfilled** |
| **/F22/** | *The OR-Pad system shall allow the user in the intraoperative application to mark case information and materials as "important".* | **Fulfilled** |
| **/F23/** | *The OR-Pad system shall allow the user in the intraoperative application to assign case information and materials to a certain phase.* | **Fulfilled** |
| **/F24/** | *The OR-Pad system shall allow the user in the intraoperative application to see the overview of the material-phase-assignment.* | **Fulfilled** |
| **/F25/** | *The OR-Pad system shall allow the user in the intraoperative application to filter case information and materials by category.* | **Fulfilled** |
| **/F26/** | *The OR-Pad system shall allow the user in the intraoperative application to create a new material by voice recording, text note, or camera recording and to upload files.* | **Fulfilled** |
| **/F27/** | *The OR-Pad system shall allow the user in the intraoperative application to finish an intervention.* | **Fulfilled** |
| **/F28/** | *The OR-Pad system shall allow the user in the mobile application to post-process an intervention.* | **Fulfilled** |
| **/F29/** | *The mobile OR-Pad system records user authentication data.* | **Fulfilled** |
| **/F30/** | *The OR-Pad system records new materials related to an intervention.* | **Fulfilled** |
| **/F31/** | *The OR-Pad system outputs available case information of an intervention from the HIS.* | **Fulfilled** |
| **/F32/** | *The OR-Pad system outputs materials created for an intervention from the HIS.* | **Fulfilled** |
| **/F33/** | *The OR-Pad system outputs OR information of an intervention.* | **Fulfilled** |
| **/F34/** | *The intraoperative OR-Pad system records OR registration data.* | **Fulfilled** |
| **/F35/** | *The intraoperative OR-Pad system outputs materials or case information according to the surgical phase defined by the user.* | **Fulfilled** |
| **/F36/** | *The intraoperative OR-Pad system generates and outputs the remaining time concerning the specified surgery time.* | **Fulfilled** |
| **/F37/** | *When the mobile OR-Pad system is started, it shall display appropriate input fields for logging in with the user data.* | **Fulfilled** |
| **/F38/** | *When entering the user data, the mobile OR-Pad system shall display the interventions to which the user is assigned as surgeon or assistant physician.* | **Fulfilled** |
| **/F39/** | *When installing/attaching the tablet in the OR, the intraoperative OR-Pad system shall display appropriate input fields for the registration of the OR.* | **Fulfilled** |
| **/F40/** | *When entering the access data of the OR, the intraoperative OR-Pad system shall display the next intervention taking place that is assigned to the OR.* | **Fulfilled** |
| **/F41/** | *At the start of the intervention, the intraoperative OR-Pad system shall display the case information, materials, and OR information of the intervention currently taking place or being in preparation.* | **Fulfilled** |
| **/F42/** | *When an intervention is selected, the OR-Pad system shall display the case information, OR information, and specially created materials available in the HIS.* | **Fulfilled** |
| **/F43/** | *If new case information is available in the HIS, the OR-Pad system shall reload and display it.* | **Fulfilled** |
| **/F44/** | *When selecting a material or case information, the OR-Pad system shall display it maximized.* | **Fulfilled** |
| **/F45/** | *When creating a new material, the OR-Pad system shall save it to the selected intervention.* | **Fulfilled** |
| **/F46/** | *When editing a material or case information, the OR-Pad system shall display the available tools for the selected file type.* | **Fulfilled** |
| **/F47/** | *When applying a tool to a material or case information, the OR-Pad system shall execute and display the corresponding modification.* | **Fulfilled** |
| **/F48/** | *When saving a customized material or case information, the OR-Pad system shall treat it as a newly created material.* | **Fulfilled** |
| **/F49/** | *When deleting a material, the OR-Pad system shall delete it in the HIS.* | **Fulfilled** |
| **/F50/** | *When marking case information or materials as "important", the OR-Pad system shall save this assignment.* | **Fulfilled** |
| **/F51/** | *When applying the filter, the OR-Pad system shall only display the information that corresponds to the selected categories.* | **Fulfilled** |
| **/F52/** | *When case information or materials are assigned to a specific surgical phase, the OR-Pad system shall save this assignment.* | **Fulfilled** |
| **/F53/** | *When recognizing a surgical phase, the intraoperative OR-Pad system shall display information assigned to this phase.* | **Fulfilled** |
| **/F54/** | *When the OR-Pad system is terminated, the communication to the OR-Pad server and the HIS shall be terminated and the application be closed.* | **Fulfilled** |
| **/F55/** | *A user who is assigned as a surgeon in the HIS shall be able to log into the mobile OR-Pad system, for users of other user groups the login fails.* | **Fulfilled** |
| **/F56/** | *A user who is assigned as an OR in the HIS shall be able to log into the intraoperative OR-Pad system, for users of other user groups the login fails.* | **Fulfilled** |
| **/F57/** | *If no intervention or preparation for an intervention is done at the current time, the intraoperative OR-Pad system shall not display any case information and materials.* | **Not fulfilled** |
| **/F58/** | *Materials shall only be creatable or adjustable for interventions in which the user is assigned as surgeon or assistant.* | **Fulfilled** |
| **/N01/** | *The information provided by the OR-Pad system shall serve as a memory aid to support the surgeon (non-diagnostic visualization).* | **Fulfilled** |
| **/N02/** | *The functions provided by the tools of the OR-Pad system shall be functional for demonstration purposes.* | **Fulfilled** |
| **/N03/** | *In addition to an exemplary connection to a HIS, the OR-Pad system shall also be functional without this with local dummy data for demonstration purposes.* | **Not fulfilled** |
| **/N04/** | *The OR-Pad system shall use standardized protocols for communication and data exchange with other systems.* | **Fulfilled** |
| **/N05/** | *User data shall be kept confidential by the OR-Pad system.* | **Not fulfilled** |
| **/N06/** | *It shall be possible to demonstrate the functions of the OR-Pad system.* | **Fulfilled** |
| **/N07/** | *The OR-Pad system shall catch errors and output meaningful error messages in the event of a failure or invalid input.* | **Fulfilled** |
| **/N08/** | *The OR-Pad system shall inform the user of restrictions for data inputs.* | **Fulfilled** |
| **/N09/** | *The OR-Pad system shall be recoverable by rebooting.* | **Fulfilled** |
| **/N10/** | *The OR-Pad system shall be understandable and operable without assistance.* | **Fulfilled** |
| **/N11/** | *After using the mobile OR-Pad system, users shall be able to use the intraoperative OR-Pad system.* | **Fulfilled** |
| **/N12/** | *The OR-Pad system shall be operable in the OR by the surgeon or the sterile assistant.* | **Partially fulfilled** |
| **/N13/** | *The user interfaces of the OR-Pad system shall make it possible to achieve desired functions and displays with a minimum of time.* | **Fulfilled** |
| **/N14/** | *The user interfaces of the OR-Pad system shall appear modern and professional.* | **Fulfilled** |
| **/N15/** | *The applications of the OR-Pad system shall answer user queries without visible delay.* | **Partially fulfilled** |
| **/N16/** | *The tablet of the OR-Pad system in the OR shall have a continuous power supply in the holder.* | **Partially fulfilled** |
| **/N17/** | *The OR-Pad system shall be modular so that it can be expanded to include additional functions.* | **Fulfilled** |
| **/N18/** | *The integration of additional functions shall not affect the basic functionality of the OR-Pad system.* | **Fulfilled** |
| **/N19/** | *Functional tests for manual execution shall be described to check the functionalities of the OR-Pad system.* | **Not fulfilled** |
| **/N20/** | *The program code of the OR-Pad system, both server- and client-side, shall have code documentation.* | **Fulfilled** |
| **/N21/** | *The system architecture and the communication between server and client shall be recorded in a document.* | **Fulfilled** |
| **/N22/** | *The OR-Pad system shall be connectable to different HIS.* | **Not fulfilled** |
| **/N23/** | *The OR-Pad system shall be usable for different types of interventions.* | **Fulfilled** |
| **/N24/** | *The OR-Pad system shall be adjustable in height and orientation to the needs of the surgeon in the OR.* | **Not verified** |
| **/N25/** | *The OR-Pad system shall be installable by a member of the IT department.* | **Not fulfilled** |
| **/N26/** | *The OR-Pad system shall not cause any data loss in the HIS.* | **Fulfilled** |
