# Supplementary material for: A high-fidelity prototype of a sterile information system for the perioperative area: OR-Pad
Source: Int J Comput Assist Radiol Surg. 2022 Nov 12;18(3):575–85. doi: 10.1007/s11548-022-02787-w (PMC9939502; doi:10.1007/s11548-022-02787-w)
Supplement: Supplementary file 2 — Supplementary file2 (DOCX 41 kb) [file 11548_2022_2787_MOESM2_ESM.docx]

**Supplementary Material for the article “A high-fidelity prototype of a sterile information system for the perioperative area - OR-Pad” published in the "International Journal of Computer Assisted Radiology and Surgery"**

C. Ryniak¹*, S. M. Frommer¹*, D. Junger¹, S. Lohmann¹, M. Stadelmaier¹, P. Schmutz¹, A. Stenzl^2^, B. Hirt^3^, O. Burgert¹

¹Reutlingen University, School of Informatics, Research Group Computer Assisted Medicine (CaMed) Reutlingen, Germany

^2^University Hospital Tübingen, Department of Urology, Tübingen, Germany

^3^Eberhard Karls University Tübingen, Faculty of Medicine, Department of Anatomy, Institute for Clinical Anatomy and Cell Analytics, Tübingen, Germany

*The first two authors contributed equally to this paper.

**Corresponding Author**

Denise Junger, ORCID: 0000-0002-7895-3210

Reutlingen University, School of Informatics, Research Group Computer Assisted Medicine (CaMed), Reutlingen, Germany

Contact: denise.junger@reutlingen-university.de, Tel.: +49 (0)7121 – 271 4090

**Online Resource 2: Requirements analysis & Functional evaluation (detailed version)**

**Table 2** Requirements analysis & Functional evaluation of the *OR-Pad* system (detailed version). V = Vision, G = Goal, F = Functional requirement, N = Non-functional requirement, OR = Operating room, HIS = Hospital information system. As the primary evaluation method, the *OR-Pad* demo prototype was executed by the developers and the requirements contrasted with the actual functionalities of the prototype. Code analysis was used in addition for intern details. Usability tests with clinicians were conducted in specific cases (e.g. to evaluate fast access to information).

| ***No.*** | ***Vision, goal or (non-) functional requirement*** | ***Evaluation method*** | ***Evaluation result*** |
| --- | --- | --- | --- |
| **/V01/** | *The OR-Pad system offers fast, uncomplicated access to available case information of an intervention.* | Usability test: Fast access to case information without any complications, information was quickly found via the timeline | **Fulfilled** |
| **/V02/** | *The OR-Pad system enables the creation and facilitates the transfer of materials into and out of the OR.* | Mobile and intraoperative mode which share the same concept and information base | **Fulfilled** |
| **/V03/** | *The OR-Pad system displays materials and case information close by the surgeon during an intervention.* | Open issues for holding arm and sterility concept | **Partially fulfilled** |
| **/V04/** | *The OR-Pad system allows to view materials and case information and to highlight relevant aspects in them.* | Timeline overview of all information and edit mode for highlighting purposes | **Fulfilled** |
| **/V05/** | *The OR-Pad system supports the surgeon during an intervention with context-relevant information.* | Demo of a situation recognition provides the surgical phase and delay, the phase is used to suggest assigned material | **Fulfilled** |
| **/G01/** | *To provide uncomplicated access to available case information of an intervention, the OR-Pad system should realize a connection to various HIS and thus enable bundled access to the available case information.* | Demo of a HIS | **Fulfilled** |
| **/G02/** | *To provide quick access to available case information of an intervention, the OR-Pad system should only display information related to the case and thus reduce the amount of data.* | Case information is requested from the HIS for a specific case | **Fulfilled** |
| **/G03/** | *To facilitate taking materials into the OR, the OR-Pad system should provide a mobile application for preparation that allows files, such as notes, sketches, voice recordings, etc., to be added and saved for later use.* | Mobile mode for preparation to add notes, images, or voice recordings and edit information | **Fulfilled** |
| **/G04/** | *To facilitate the taking of materials out of the OR, the OR-Pad system should provide a mobile application on a tablet during the intervention that allows the creation and storage of files, such as camera screenshots, sketches, voice recordings, etc., for later use.* | Intraoperative mode to add notes, images, or voice recordings and edit information | **Fulfilled** |
| **/G05/** | *To enable the creation of materials, the OR-Pad system should provide tools for voice recordings, text notes, and camera recordings, as well as allow to upload files to provide an appropriate form of input depending on the situation.* | Hardware access to tablet/phone | **Fulfilled** |
| **/G06/** | *To display materials and case information near the surgeon during an intervention, the OR-Pad system should be mounted in the vicinity of the OR site via a suitable holding construction and thus be operable by the surgeon.* | Open issues for holding arm and sterility concept | **Partially fulfilled** |
| **/G07/** | *To enable a view of materials and case information, the OR-Pad system should include appropriate tools to display this data, making it accessible to the user.* | Timeline overview and detailed view of all information | **Fulfilled** |
| **/G08/** | *To enable highlighting of relevant aspects in materials and case information, the OR-Pad system should provide appropriate tools for modification, making the view of the data changeable for the user.* | Edit mode to sketch into a material or case information | **Fulfilled** |
| **/G09/** | *To support the surgeon with context-relevant information during an intervention, the OR-Pad system should offer the user the possibility to determine which material or case information should be displayed at which surgical phase.* | Demo of a situation recognition, materials can be assigned to surgical phases | **Fulfilled** |
| **/G10/** | *To support the surgeon with context-relevant information during an intervention, the OR-Pad system should visualize the temporal progress and thus provide the user with feedback on whether the schedule can be adhered to.* | Demo of a situation recognition provides the delay | **Fulfilled** |
| **/F01/** | *The OR-Pad system shall allow the user in the mobile application to authenticate himself.* | Login | **Fulfilled** |
| **/F02/** | *The OR-Pad system shall allow the user in the mobile application to select an intervention.* | Intervention overview, an intervention can be selected for further information | **Fulfilled** |
| **/F03/** | *The OR-Pad system shall allow the user in the mobile application to prepare an intervention.* | Information for a selected intervention can be edited | **Fulfilled** |
| **/F04/** | *The OR-Pad system shall allow the user in the mobile application to select a case information or material.* | A material or case information can be viewed in full-screen | **Fulfilled** |
| **/F05/** | *The OR-Pad system shall allow the user in the mobile application to view case information and materials.* | See **/F04/** | **Fulfilled** |
| **/F06/** | *The OR-Pad system shall allow the user in the mobile application to select tools to edit the case information and materials.* | Edit mode enables sketching into the information | **Fulfilled** |
| **/F07/** | *The OR-Pad system shall allow the user in the mobile application to customize case information and materials with the provided tools.* | See **/F06/** | **Fulfilled** |
| **/F08/** | *The OR-Pad system shall allow the user in the mobile application to mark case information and materials as "important".* | Star icon | **Fulfilled** |
| **/F09/** | *The OR-Pad system shall allow the user in the mobile application to assign case information and materials to a certain phase.* | Demo of a situation recognition (see **/G09/**) | **Fulfilled** |
| **/F10/** | *The OR-Pad system shall allow the user in the mobile application to see the overview of the material-phase-assignment.* | Demo of a situation recognition, material-phase-assignment is stored an can be displayed | **Fulfilled** |
| **/F11/** | *The OR-Pad system shall allow the user in the mobile application to filter case information and materials by category.* | Filter mode | **Fulfilled** |
| **/F12/** | *The OR-Pad system shall allow the user in the mobile application to create a new material via a voice recording, text note, or camera recording and to upload files.* | See **/G05/** | **Fulfilled** |
| **/F13/** | *The OR-Pad system shall allow the user in the mobile application to log out.* | Logout option | **Fulfilled** |
| **/F14/** | *The OR-Pad system shall allow the user in the intraoperative application to authenticate with the OR credentials.* | Login | **Fulfilled** |
| **/F15/** | *The OR-Pad system shall allow the user in the intraoperative application to register the tablet for the corresponding OR in which it is to be attached.* | OR is identified by login | **Fulfilled** |
| **/F16/** | *The OR-Pad system shall allow the user in the intraoperative application to log out.* | Logout option | **Fulfilled** |
| **/F17/** | *The OR-Pad system shall allow the user in the intraoperative application to start an intervention.* | Single intervention overview with start button | **Fulfilled** |
| **/F18/** | *The OR-Pad system shall allow the user in the intraoperative application to select a case information or material.* | See **/F04/** | **Fulfilled** |
| **/F19/** | *The OR-Pad system shall allow the user in the intraoperative application to view case information and materials.* | See **/F04/** | **Fulfilled** |
| **/F20/** | *The OR-Pad system shall allow the user in the intraoperative application to select tools to edit the case information and materials.* | See **/F06/** | **Fulfilled** |
| **/F21/** | *The OR-Pad system shall allow the user in the intraoperative application to customize case information and materials with the provided tools.* | See **/F06/** | **Fulfilled** |
| **/F22/** | *The OR-Pad system shall allow the user in the intraoperative application to mark case information and materials as "important".* | See **/F08/** | **Fulfilled** |
| **/F23/** | *The OR-Pad system shall allow the user in the intraoperative application to assign case information and materials to a certain phase.* | Demo of a situation recognition (see **/G09/**) | **Fulfilled** |
| **/F24/** | *The OR-Pad system shall allow the user in the intraoperative application to see the overview of the material-phase-assignment.* | Demo of a situation recognition (see **/F10/**) | **Fulfilled** |
| **/F25/** | *The OR-Pad system shall allow the user in the intraoperative application to filter case information and materials by category.* | See **/F11/** | **Fulfilled** |
| **/F26/** | *The OR-Pad system shall allow the user in the intraoperative application to create a new material by voice recording, text note, or camera recording and to upload files.* | See **/G05/** | **Fulfilled** |
| **/F27/** | *The OR-Pad system shall allow the user in the intraoperative application to finish an intervention.* | Finish option | **Fulfilled** |
| **/F28/** | *The OR-Pad system shall allow the user in the mobile application to post-process an intervention.* | Same mode as for the preparation | **Fulfilled** |
| **/F29/** | *The mobile OR-Pad system records user authentication data.* | Management of users and assignments | **Fulfilled** |
| **/F30/** | *The OR-Pad system records new materials related to an intervention.* | Storage of added or edited materials | **Fulfilled** |
| **/F31/** | *The OR-Pad system outputs available case information of an intervention from the HIS.* | Demo of a HIS stores dummy case information | **Fulfilled** |
| **/F32/** | *The OR-Pad system outputs materials created for an intervention from the HIS.* | Demo of a HIS stores dummy materials | **Fulfilled** |
| **/F33/** | *The OR-Pad system outputs OR information of an intervention.* | Demo of a HIS stores dummy surgical information | **Fulfilled** |
| **/F34/** | *The intraoperative OR-Pad system records OR registration data.* | See **/F29/** | **Fulfilled** |
| **/F35/** | *The intraoperative OR-Pad system outputs materials or case information according to the surgical phase defined by the user.* | Demo of a situation recognition, material-phase-assignment is used to suggest the assigned material if the appropriate phase is provided by the situation recognition | **Fulfilled** |
| **/F36/** | *The intraoperative OR-Pad system generates and outputs the remaining time concerning the specified surgery time.* | Demo of a situation recognition (see **/G10/**) | **Fulfilled** |
| **/F37/** | *When the mobile OR-Pad system is started, it shall display appropriate input fields for logging in with the user data.* | Login view | **Fulfilled** |
| **/F38/** | *When entering the user data, the mobile OR-Pad system shall display the interventions to which the user is assigned as surgeon or assistant physician.* | Demo HIS stores user assignments | **Fulfilled** |
| **/F39/** | *When installing/attaching the tablet in the OR, the intraoperative OR-Pad system shall display appropriate input fields for the registration of the OR.* | See **/F37/** | **Fulfilled** |
| **/F40/** | *When entering the access data of the OR, the intraoperative OR-Pad system shall display the next intervention taking place that is assigned to the OR.* | Demo OR, an intervention plan is simulated | **Fulfilled** |
| **/F41/** | *At the start of the intervention, the intraoperative OR-Pad system shall display the case information, materials, and OR information of the intervention currently taking place or being in preparation.* | Demo OR, all data from the intervention is requested depending on the intervention plan | **Fulfilled** |
| **/F42/** | *When an intervention is selected, the OR-Pad system shall display the case information, OR information, and specially created materials available in the HIS.* | Demo of a HIS (see **/F31/**, **/F32/** and **/F33/**) | **Fulfilled** |
| **/F43/** | *If new case information is available in the HIS, the OR-Pad system shall reload and display it.* | Demo of a HIS, OR-Pad automatically reloads data from the HIS | **Fulfilled** |
| **/F44/** | *When selecting a material or case information, the OR-Pad system shall display it maximized.* | Full-screen view after selection | **Fulfilled** |
| **/F45/** | *When creating a new material, the OR-Pad system shall save it to the selected intervention.* | Association of all new or edited information to the selected intervention | **Fulfilled** |
| **/F46/** | *When editing a material or case information, the OR-Pad system shall display the available tools for the selected file type.* | See **/G08/** | **Fulfilled** |
| **/F47/** | *When applying a tool to a material or case information, the OR-Pad system shall execute and display the corresponding modification.* | Live sketching (see **/G08/**) | **Fulfilled** |
| **/F48/** | *When saving a customized material or case information, the OR-Pad system shall treat it as a newly created material.* | Edits are stored as a separate file so the original will not be modified | **Fulfilled** |
| **/F49/** | *When deleting a material, the OR-Pad system shall delete it in the HIS.* | Demo of a HIS, just own materials can be deleted | **Fulfilled** |
| **/F50/** | *When marking case information or materials as "important", the OR-Pad system shall save this assignment.* | Management of the assignment to “important” | **Fulfilled** |
| **/F51/** | *When applying the filter, the OR-Pad system shall only display the information that corresponds to the selected categories.* | Filter only shows selected categories | **Fulfilled** |
| **/F52/** | *When case information or materials are assigned to a specific surgical phase, the OR-Pad system shall save this assignment.* | Demo of a situation recognition, material-phase-assignment is stored | **Fulfilled** |
| **/F53/** | *When recognizing a surgical phase, the intraoperative OR-Pad system shall display information assigned to this phase.* | Demo of a situation recognition (see **/F35/**) | **Fulfilled** |
| **/F54/** | *When the OR-Pad system is terminated, the communication to the OR-Pad server and the HIS shall be terminated and the application be closed.* | Communications are terminated by closing the application | **Fulfilled** |
| **/F55/** | *A user who is assigned as a surgeon in the HIS shall be able to log into the mobile OR-Pad system, for users of other user groups the login fails.* | Demo user, login is only possible for assigned users | **Fulfilled** |
| **/F56/** | *A user who is assigned as an OR in the HIS shall be able to log into the intraoperative OR-Pad system, for users of other user groups the login fails.* | Demo user (see **/F55/**) | **Fulfilled** |
| **/F57/** | *If no intervention or preparation for an intervention is done at the current time, the intraoperative OR-Pad system shall not display any case information and materials.* | No simulation of the temporal procedure in the OR | **Not fulfilled** |
| **/F58/** | *Materials shall only be creatable or adjustable for interventions in which the user is assigned as surgeon or assistant.* | Demo user (see **/F55/**) | **Fulfilled** |
| **/N01/** | *The information provided by the OR-Pad system shall serve as a memory aid to support the surgeon (non-diagnostic visualization).* | Provision of existing and new information but not for diagnostic purposes | **Fulfilled** |
| **/N02/** | *The functions provided by the tools of the OR-Pad system shall be functional for demonstration purposes.* | Prototypical implementation | **Fulfilled** |
| **/N03/** | *In addition to an exemplary connection to a HIS, the OR-Pad system shall also be functional without this with local dummy data for demonstration purposes.* | Just functional with the Demo HIS | **Not fulfilled** |
| **/N04/** | *The OR-Pad system shall use standardized protocols for communication and data exchange with other systems.* | FHIR for HIS communication, DICOMweb for PACS communication | **Fulfilled** |
| **/N05/** | *User data shall be kept confidential by the OR-Pad system.* | Demo user administration, no security concept yet | **Not fulfilled** |
| **/N06/** | *It shall be possible to demonstrate the functions of the OR-Pad system.* | Executable system, demonstration of pre-, intra-, and postoperative use via prototypical implementation | **Fulfilled** |
| **/N07/** | *The OR-Pad system shall catch errors and output meaningful error messages in the event of a failure or invalid input.* | Feedback via message boxes if false user inputs (e.g. wrong login, file too big for upload) or relevant information (e.g. data is loading), errors are catched on server-side, logging for debugging | **Fulfilled** |
| **/N08/** | *The OR-Pad system shall inform the user of restrictions for data inputs.* | Only supported data types are selectable, feedback via message boxes (see **/N07/**) | **Fulfilled** |
| **/N09/** | *The OR-Pad system shall be recoverable by rebooting.* | Manual start-up of all system components | **Fulfilled** |
| **/N10/** | *The OR-Pad system shall be understandable and operable without assistance.* | Usability test: Usage of the system without any assistance after a short introduction | **Fulfilled** |
| **/N11/** | *After using the mobile OR-Pad system, users shall be able to use the intraoperative OR-Pad system.* | Mobile and intraoperative mode have hardly any differences | **Fulfilled** |
| **/N12/** | *The OR-Pad system shall be operable in the OR by the surgeon or the sterile assistant.* | See **/G06/** | **Partially fulfilled** |
| **/N13/** | *The user interfaces of the OR-Pad system shall make it possible to achieve desired functions and displays with a minimum of time.* | Usability test: Function or information can be reached within a short time and a minimum of clicks | **Fulfilled** |
| **/N14/** | *The user interfaces of the OR-Pad system shall appear modern and professional.* | Usability test: The design of the graphical user interface is good | **Fulfilled** |
| **/N15/** | *The applications of the OR-Pad system shall answer user queries without visible delay.* | Delays by loading a lot of information at the same time (e.g. all information for a case) | **Partially fulfilled** |
| **/N16/** | *The tablet of the OR-Pad system in the OR shall have a continuous power supply in the holder.* | See **/G06/** | **Partially fulfilled** |
| **/N17/** | *The OR-Pad system shall be modular so that it can be expanded to include additional functions.* | Expandability with new modules and functionalities | **Fulfilled** |
| **/N18/** | *The integration of additional functions shall not affect the basic functionality of the OR-Pad system.* | The modular structure enables easy expandability (see **/N17/**) | **Fulfilled** |
| **/N19/** | *Functional tests for manual execution shall be described to check the functionalities of the OR-Pad system.* | No described functional tests | **Not fulfilled** |
| **/N20/** | *The program code of the OR-Pad system, both server- and client-side, shall have code documentation.* | Comments in the code | **Fulfilled** |
| **/N21/** | *The system architecture and the communication between server and client shall be recorded in a document.* | Documentation of the concept and prototypical implementation | **Fulfilled** |
| **/N22/** | *The OR-Pad system shall be connectable to different HIS.* | Demo of a HIS (see **/N03/**) | **Not fulfilled** |
| **/N23/** | *The OR-Pad system shall be usable for different types of interventions.* | Demonstration for seven different use cases | **Fulfilled** |
| **/N24/** | *The OR-Pad system shall be adjustable in height and orientation to the needs of the surgeon in the OR.* | See **/G06/** | **Not verified** |
| **/N25/** | *The OR-Pad system shall be installable by a member of the IT department.* | Local operation | **Not fulfilled** |
| **/N26/** | *The OR-Pad system shall not cause any data loss in the HIS.* | No data loss detected | **Fulfilled** |
